# Exposure to Fluoride in Drinking Water and Hip Fracture Risk: A Meta-Analysis of Observational Studies

**DOI:** 10.1371/journal.pone.0126488

**Published:** 2015-05-28

**Authors:** Xin-Hai Yin, Guang-Lei Huang, Du-Ren Lin, Cheng-Cheng Wan, Ya-Dong Wang, Ju-Kun Song, Ping Xu

**Affiliations:** Department of Oral and Maxillary Surgery, Gui Zhou provincial people's hospital, Guiyang, Gui Zhou, PR China; Harvard Medical School, UNITED STATES

## Abstract

**Background:**

Many observational studies have shown that exposure to fluoride in drinking water is associated with hip fracture risk. However, the findings are varied or even contradictory. In this work, we performed a meta-analysis to assess the relationship between fluoride exposure and hip fracture risk.

**Methods:**

PubMed and EMBASE databases were searched to identify relevant observational studies from the time of inception until March 2014 without restrictions. Data from the included studies were extracted and analyzed by two authors. Summary relative risks (RRs) with corresponding 95% confidence intervals (CIs) were pooled using random- or fixed-effects models as appropriate. Sensitivity analyses and meta-regression were conducted to explore possible explanations for heterogeneity. Finally, publication bias was assessed.

**Results:**

Fourteen observational studies involving thirteen cohort studies and one case-control study were included in the meta-analysis. Exposure to fluoride in drinking water does not significantly increase the incidence of hip fracture (RRs, 1.05; 95% CIs, 0.96–1.15). Sensitivity analyses based on adjustment for covariates, effect measure, country, sex, sample size, quality of Newcastle–Ottawa Scale scores, and follow-up period validated the strength of the results. Meta-regression showed that country, gender, quality of Newcastle–Ottawa Scale scores, adjustment for covariates and sample size were not sources of heterogeneity. Little evidence of publication bias was observed.

**Conclusion:**

The present meta-analysis suggests that chronic fluoride exposure from drinking water does not significantly increase the risk of hip fracture. Given the potential confounding factors and exposure misclassification, further large-scale, high-quality studies are needed to evaluate the association between exposure to fluoride in drinking water and hip fracture risk.

## Background

Hip fractures, or fractures of the femoral neck, are a major public health problem and are the leading cause of morbidity and mortality in people aged ≥65 years in many countries. Hip fracture is also one of the most common causes of admission to nursing homes. An estimated 1.7 million hip fractures occurred worldwide in the year 1990 [1]. The number of people sustaining a hip fracture continues to rise because of an increasing elderly population. Hip fractures also pose a substantial challenge to both patients and healthcare systems worldwide. Currently, over 320,000 hip fractures occur in North America alone each year, and this number is expected to rise to 580,000 by 2040 with healthcare costs exceeding 10 billion dollars [2, 3]. Thus, factors related to hip fracture development need better understanding.

Considering our widespread exposure to fluoride in drinking water, the effect of fluoride on hip fracture risk is an important public health issue. Fluoride in drinking water derives from natural sources or is added to protect dental health. People are exposed daily to fluoride through food and water. In the past 60 years, the possible adverse effects of fluoride on human health have been controversial. The cariostatic benefit from water fluoridation is indisputable. However, results from epidemiologic studies have been contradictory. Some studies have suggested a positive association between the concentration of fluoride in water and incidence of fractures [4–7], but others have found no association [8–15] or even an inverse relation [16, 17]. Therefore, the association between fluoride exposure in drinking water and hip fracture risk remains unclear. Therefore, a quantitative and systematic summary of the evidence should be performed using meta-analysis. In the present study, we conducted a meta-analysis to confirm the hypothesis whether fluoride exposure in drinking water has increased or decreased the risk of hip fracture.

## Methods

### Search strategy

We searched the PubMed and EMBASE databases (from time of inception until March 2014) to identify relevant studies that investigated the association between fluoride exposure in drinking water and hip fracture risk. The following search terms were used: “fluorides” or “fluoride” or “fluoridated” or “fluoridation” and “hip fracture”or “hip fractures” filtered by Human without language restrictions. We also reviewed the reference lists of pertinent articles and recent reviews.

### Inclusion and exclusion criteria

Studies were considered acceptable for inclusion in the meta-analysis if they met the following criteria: (1) evaluating the association between fluoride exposure in drinking water and hip fracture risk with obtainable full text; (2) providing adjusted and/or unadjusted hazard ratios (HRs), odds ratios (ORs), and relative risks (RRs) with corresponding 95% confidence interval (CI) or raw data for calculating crude HRs, ORs, or RRs. Studies were excluded if they met the following criteria: letters, comments, correspondences, conference reports, or laboratory studies; or they did not contain enough data for risk estimates calculation. If duplicated data were present in several studies, only the most recent, largest, or most comprehensive study was included. Two authors (JKS and XHY) independently evaluated the eligibility of all retrieved studies, and disagreements were resolved by discussion or consultation with a third author (GLH).

### Data extraction

Two authors (JKS and XHY) independently extracted data of the characteristics of the selected studies using a standardized data extraction form. Data were recorded as follows: first author’s surname, publication year, study design, country, number of subjects (cases/controls), gender, follow-up duration, period of fluoride exposure, crude and/or adjusted point estimates and corresponding 95% CIs for each category, and covariate features included in the multivariable model. Disagreements were resolved by discussion and consensus with a third author (GLH).

### Quality assessment

The methodological quality of each trial was evaluated using Newcastle—Ottawa Scale (NOS). Three major components were collected: selection of study groups (0 to 4 points), ascertainment for exposure of interest in the studies (0 to 3 points), and quality of adjustment for confounding factors (0 to 2 points). A higher score represented better methodological quality. The quality of each study was graded either low (0 to 4) or high (5 to 9) level.

### Statistical analysis

RRs with corresponding 95% CIs were used as common measures of association between fluoride exposure in drinking water and hip fracture risk. Given the low absolute risk of hip fracture, ORs and HRs were directly considered approximations of RRs. Six studies [7, 11, 13–15, 17] did not report overall risk estimates but separately presented results for men and women. Therefore, we combined the results using random effects and included the pooled risks estimates in the primary analysis. One study [16] reported stratified risks estimates by age and gender, and we combined these estimates using a random-effects model and then used the pooled estimates for the meta-analysis. For three studies [5, 10, 12] that reported stratified risks estimates by fluoride exposure levels, we only used the estimates for the highest versus the lowest category of fluoride exposure levels. One study [9] presented separate risks estimates for durations of exposure, and we only used the estimates for the highest versus the lowest category of fluoride exposure duration.

Statistical heterogeneity was evaluated using Cochrane Q test (significance level at <0.10). The degree of heterogeneity was quantified using I^2^ statistic, which represents the percentage of the total variability across studies [18]. Studies with an I^2^ statistic of 25% to 50% have low heterogeneity, those with 50% to 75% have moderate heterogeneity, and those with >75% have high heterogeneity. An I^2^ value > 50% indicates significant heterogeneity. Fixed-effects model was used as pooling method for moderate or low heterogeneity (I^2^ < 50%), whereas random-effects model (REM) was used for significant heterogeneity (I^2^ > 50%). Given that patient characteristics, study design, and other confounding factors were inconsistent among studies, we further conducted sensitivity analyses to explore possible explanations for heterogeneity and to examine the influence of various exclusion criteria on the overall pooled estimate. We also investigated the influence of individual studies on the overall risk estimate. We performed meta-regression to explore the sources of heterogeneity in the association between exposure to fluoride in drinking water and hip fracture risk as reported in individual studies, particularly the effects of five study-level characteristics (country, gender, quality of Newcastle—Ottawa Scale scores, adjustment for covariates and sample size). The presence of publication bias was assessed using Begg’s and Egger’s tests [19, 20]. A P value < 0.05 was considered statistically significant, unless otherwise specified. All statistical analyses were performed using STATA version 12.0 (Stata Corporation, College Station, Texas, USA).

## Results

### Study selection

**Fig 1:**
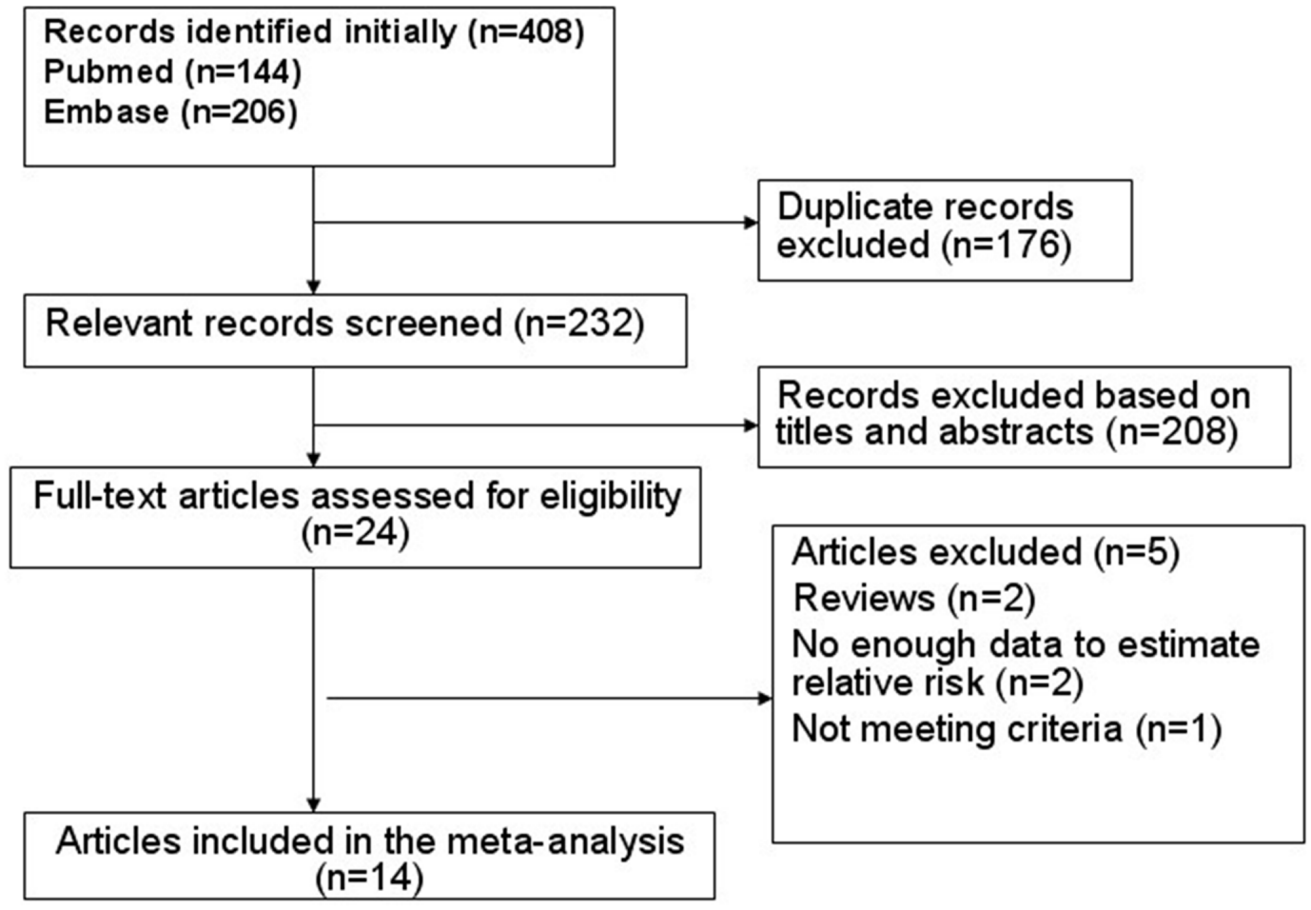
Flow chart of identification of eligible studies to final inclusion.

Upon search strategy, 408 records were initially retrieved. The majority of the retrieved articles were excluded after reviewing titles and abstracts, mainly because they were reviews, letters, comments, or irrelevant to our analysis. Twenty-four articles were considered of interest, and full text of each was retrieved for detailed evaluation. Ten out of these 24 articles were excluded. Finally, 14 articles were included in the meta-analysis (Fig 1).

### Study characteristics

The characteristics of all included articles are presented in Tables 1 and 2. Fourteen observational articles involving more than 73,411,368 individuals and 319,608 patients were identified. Among these articles, thirteen were cohort studies [4, 5, 7–17], and one was a case-control study [6]. Seven articles were based in the United States [4, 7–9, 11, 14, 15], five in Europe [5, 6, 10, 16, 17], and two in Asia [12, 13]. The articles were published from 1983 to 2013. The sample size of the observational studies ranged from 506 to 70,000,000, with the eight largest studies recruited over 100,000 participants.

**Table 1:** Characteristic of studies included in the meta-analysis.

Study	Year	Country	Study design	No. of subjects	No. of patients	Sex	Age,Median(Range),yrs	Flouride exposure (yrs)	Follow-up(yrs)	Adjustment for covariates
Simonen O	1985	Finland	Cohort study	133,398	395	F and M	NA(≥50)	19	11	Unadjust
Sowers MF	1991	United States	A prospective cohort study	506	7	F	NA(20–80)	NA	5	Adjusted for age and body size
Danielson C	1992	United States	Ecological cohort	6,049	246	F and M	NA(≥65)	20	7	Unadjust
Jacobsen SJ	1992	United States	Cohort study	70,000,000	218,951	F and M	NA(≥65)	NA	4	Adjusted for age
Jacobsen SJ	1993	United States	A retrospective-prospective cohort	NA	751	F and M	NA(≥50)	20	10	Adjusted for age.
Cauley JA	1995	United States	A prospective cohort study	2,076	41	F	NA(≥65)	12.7 ± 8.0	6.1	Adjusted for age, BMI, total calcium intake (diet plus supplements), history of osteoporosis, surgical menopause. history of kills in past year, drinks per week, education, current estrogen use. current thiazidc diuretic use, ever used bottled water.
Karagas MR	1996	United States	A retrospective cohort study	444,000	28,194	F and M	NA(65–89)	NA	4	Unadjust
Lehmann R	1998	Germany	A retrospective cohort	623,602	1,198	F and M	NA(≥35)	7–17	2	Unadjust
Kurttio P	1999	Finland	A retrospective cohort study	144,627	4,449	F and M	NA(50–80)	NA	13	Adjusted for age and area
Phipps KR	2000	United States	Multicentre prospective cohort study	7,129	231	F	74.2(≥65)	44	7	Adjusted for age, weight, education, muscle strength, surgical menopause, calcium intake, drinks/week, current oestrogen use, current thiazide use, noninsulin dependent diabetes, current thyroid hormone use, walking for exercise, and smoking status
Hillier S	2000	United Kingdom	Case-control study	1,041	514	F and M	NA(≥50)	NA	NA	Adjusted for age and gender、all potential confounders
Li Y	2001	china	Cohort study	8266	56	F and M	62.8(NA)	>25	NA	Unadjust
Park EY	2008	Korean	Cohort study	1,567,397	3,842	F and M	NA(≥65)	NA	7	Unadjust
Näsman P	2013	Sweden	Cohort study	473,277	60,733	F and M	62.8(44.0–87.0)	NA	16.8	Adjusted for gender, age group, county of residence, calendar group

F, female; M, male; NA, not available.

**Table 2:** The effect size across included studies.

Study	Year	exposure to waterfluoridation	RRs/HRs/ORs (95%CIs)
Simonen O	1985	1.0 vs. 0–0.1mg/l	
		female	1.5(1.2 to 1.8)
		male	2.5(1.6 to 3.9)
Sowers MF	1991	4.0±0.1 vs. 1.0 mg/l	2.2(1.1 to 4.7)
Danielson C	1992	1.0 vs. 0.3ppm	
		female	1.27(1.08 to 1.46)
		male	1.41(1.0 to 1.81)
Jacobsen SJ	1992	nonfluoradated vs. fluoridated	
		female	1.17(1.13 to 1.22)
		male	1.08(1.06 to 1.1)
Jacobsen SJ	1993	nonfluoradated vs. fluoridated	
		male	0.78(0.37 to 1.66)
		female	0.6(0.42 to 0.85)
Cauley JA	1995	1.01±0.21 vs. 0.15±0.10 mg/l	
		0 year	1.0
		1–10 years	0.89(0.42 to 1.92)
		11–20years	0.58(0.14 to 2.48)
		>20 years	0.44(0.1 to 1.86)
Karagas MR	1996	nonfluoradated vs. fluoridated	
		male	1(0.92 to 1.09)
		female	1.01(0.96 to 1.06)
Lehmann R	1998	0.77–1.20 vs. 0.08–0.36mg/l	
		male	
		60–64 years	2.14(0.89 to 5.2)
		65–69 years	0.55(0.22 to 1.39)
		70–74 years	0.78(0.27 to 1.39)
		75–79 years	1.05(0.65 to 1.69)
		80–84 years	1.02(0.67 to 1.55)
		≥80 years	1.92(1.07 to 3.45)
		female	
		60–64 years	0.9(0.51 to 1.58)
		65–69 years	1.56(1 to 2.44)
		70–74 years	1.09(0.76 to 1.57)
		75–79 years	1.38(1.06 to 1.8)
		80–84 years	1.2(0.95 to 1.52)
		≥80 years	1.41(1.1 to 1.81)
Kurttio P	1999	female	
		≤0.10 mg/l	1.0
		0.11–0.30 mg/l	0.93(0.84 to 1.02)
		0.31–0.50 mg/l	1.12(0.93 to 1.34)
		0.51–1.00 mg/l	1.12(0.96 to 1.31)
		1.10–1.50 mg/l	1.08(0.88 to 1.32)
		>1.50 mg/l	1.08(0.8 to 1.46)
		male	
		≤0.10 mg/l	1.0
		0.11–0.30 mg/l	1.05(0.9 to 1.22)
		0.31–0.50 mg/l	0.72(0.51 to 1.02)
		0.51–1.00 mg/l	1.03(0.81 to 1.32)
		1.10–1.50 mg/l	0.67(0.46 to 0.97)
		>1.50 mg/l	0.98(0.61 to 1.6)
Phipps KR	2000	mixed exposure	0.73(0.49 to 1.09)
		continuous exposure	0.69(0.5 to 0.96)
Hillier S	2000	≥0.9 vs. <0.9mg/l	1(0.7 to 1.5)
Li Y	2001	0.25–0.34 mg/l	0.99(0.21 to 4.77)
		0.58–0.73 mg/l	1.12(0.35 to 3.62)
		1.00–1.06 mg/l	1.0
		1.45–2.19 mg/l	2.13(0.76 to 5.96)
		2.62–3.56 mg/l	1.73(0.56 to 5.33)
		4.32–7.97 mg/l	3.26(1.20 to 8.82)
Park EY	2008	fluoridated vs. nonfluoradated	
		female	0.88(0.77 to 1)
		male	0.91(0.84 to 0.99)
Näsman P	2013	Very low <0.3	1.0
		Low 0.3–0.69	0.97(0.94 to 0.99)
		Mendium 0.7–1.49	0.97(0.94 to 1)
		High ≥1.5	0.98(0.93 to 1.04)

All 14 studies reported exposure to fluoride in drinking water. Ten studies were designed to evaluate the RRs of hip fracture [4, 5, 7–9, 11, 13–15, 17], three were designed to evaluate the ORs of hip fracture [6, 12, 16], and one was designed to evaluate the HRs of hip fracture [10]. Three studies investigated only women [4, 8, 9], whereas eleven studies investigated both women and men [5–7, 10–17]. The average follow-up ranged from 2 years to 16.8 years. Patients were followed up over five years in majority of the studies (64.3%). The association between exposure to water fluoridation and hip fracture risk was the primary outcome of interest for eight studies [5–7, 10, 13–15, 17], whereas this association was a secondary subject in six studies [4, 8, 9, 11, 12, 16]. Six studies did not adjust for confounding factors [7, 11–13, 16, 17], whereas the others controlled a group of conventional risk factors for hip fracture, such as age, gender, area, and smoking [4–6, 8–10, 14, 15].

The quality of the included studies was assessed by NOS (Table 3). The median NOS score was 6.5 (range: 4 to 9).

**Table 3:** Quality assessment of included studies based on Newcastle-Ottawa scale.

Author	year	Selection	Comparability	Exposure
Simonen O	1985	3	0	2
Sowers MF	1991	3	1	2
Danielson C	1992	3	1	3
Jacobsen SJ	1992	2	1	2
Jacobsen SJ	1993	3	1	1
Cauley JA	1995	3	2	2
Karagas MR	1996	3	0	2
Lehmann R	1998	3	0	1
Kurttio P	1999	4	1	3
Phipps KR	2000	3	2	2
Hillier S	2000	3	2	2
Li Y	2001	3	2	1
Park EY	2008	3	2	2
Näsman P	2013	4	2	3

### Exposure to fluoride in drinking water and hip fracture risk

**Fig 2:**
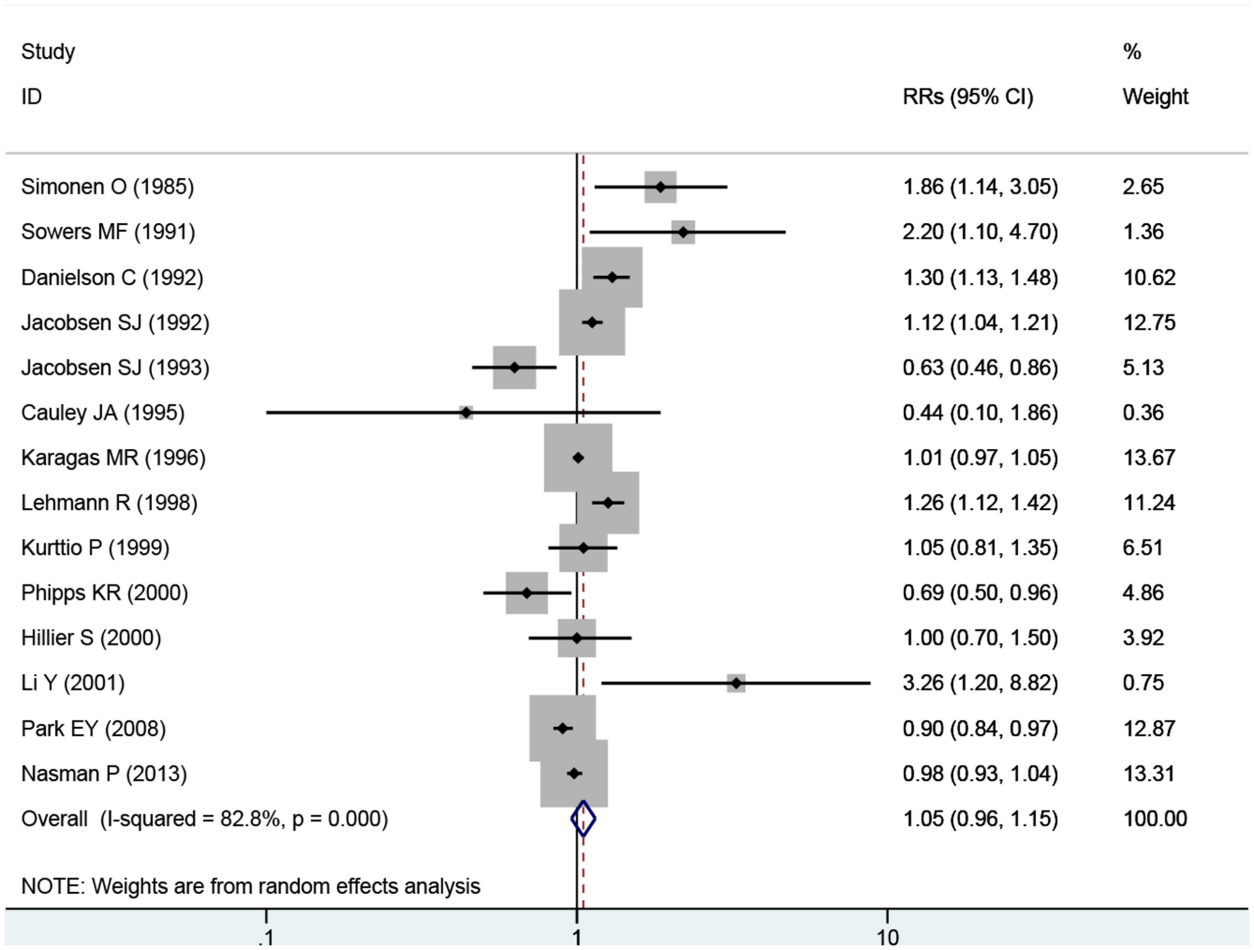
Forest plot of exposure to fluoride in the drinking water and hip fracture risk. Studies are pooled with a random-effects model.

The overall RR estimates for each study were pooled to determine the total estimates of risk using random-effects model (RRs = 1.05; 95% CIs = 0.96 to 1.15, P = 0.291), and the heterogeneity was significant (P < 0.001, I^2^ = 82.8%). The results suggested that, exposure to fluoride in drinking water does not increase the incidence of hip fracture risk and substantial heterogeneity was observed (Fig 2).

**Fig 3:**
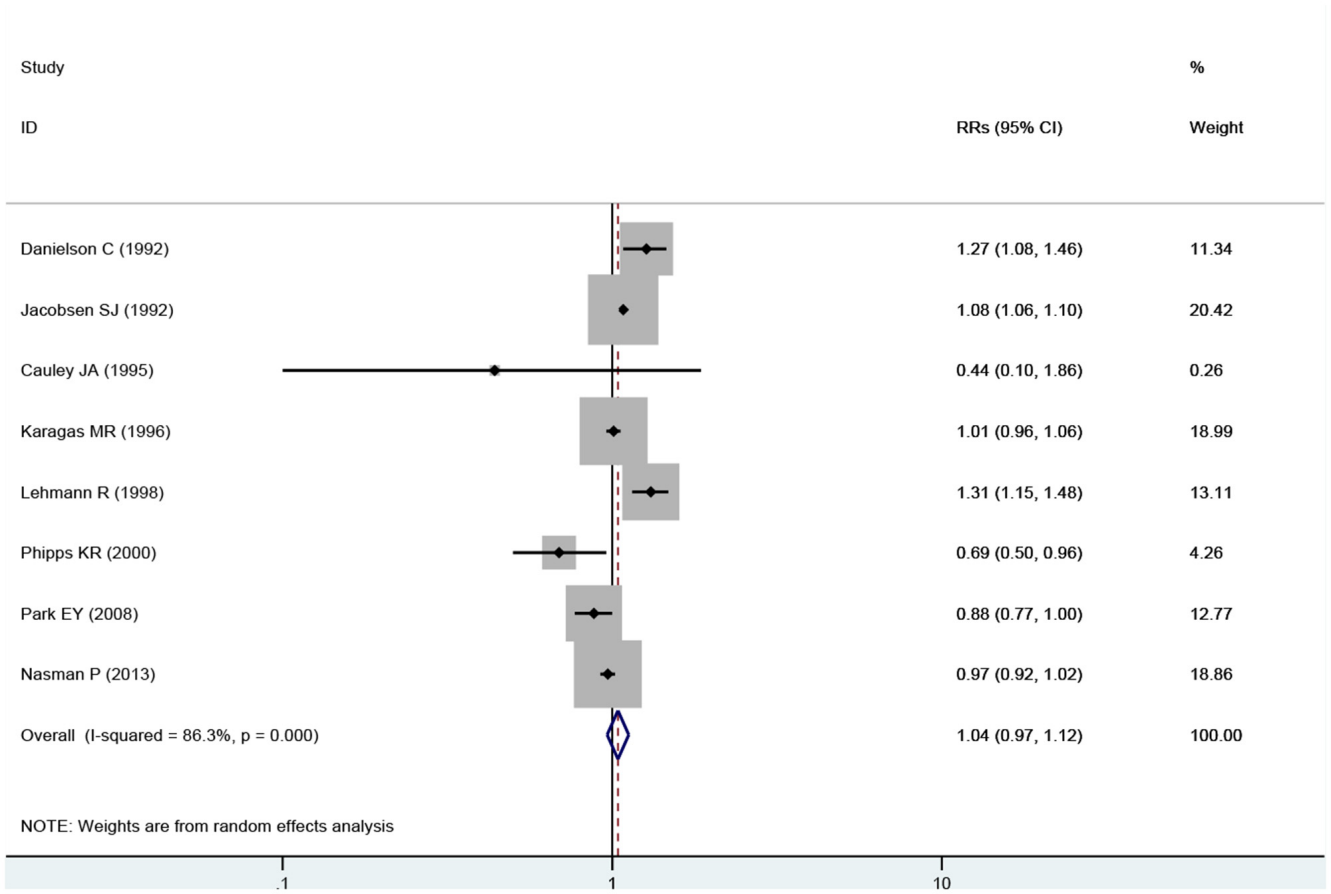
Forest plot of exposure to fluoride in the drinking water and hip fracture risk among women beyond 65 years. Studies are pooled with a random-effects model.

We also conducted meta-analyses based on women aged ≥65 years to explore the effect of exposure to fluoride from drinking water on hip fracture risk, and the results were relatively consistent. No association was observed in exposure to fluoride from drinking water on hip fracture risk (RRs = 1.04, 95% CIs = 0.97 to 1.12, P = 0.30) with substantial evidence of heterogeneity (P < 0.001, I^2^ = 86.3%) (Fig 3).

### Sensitivity analyses

**Fig 4:**
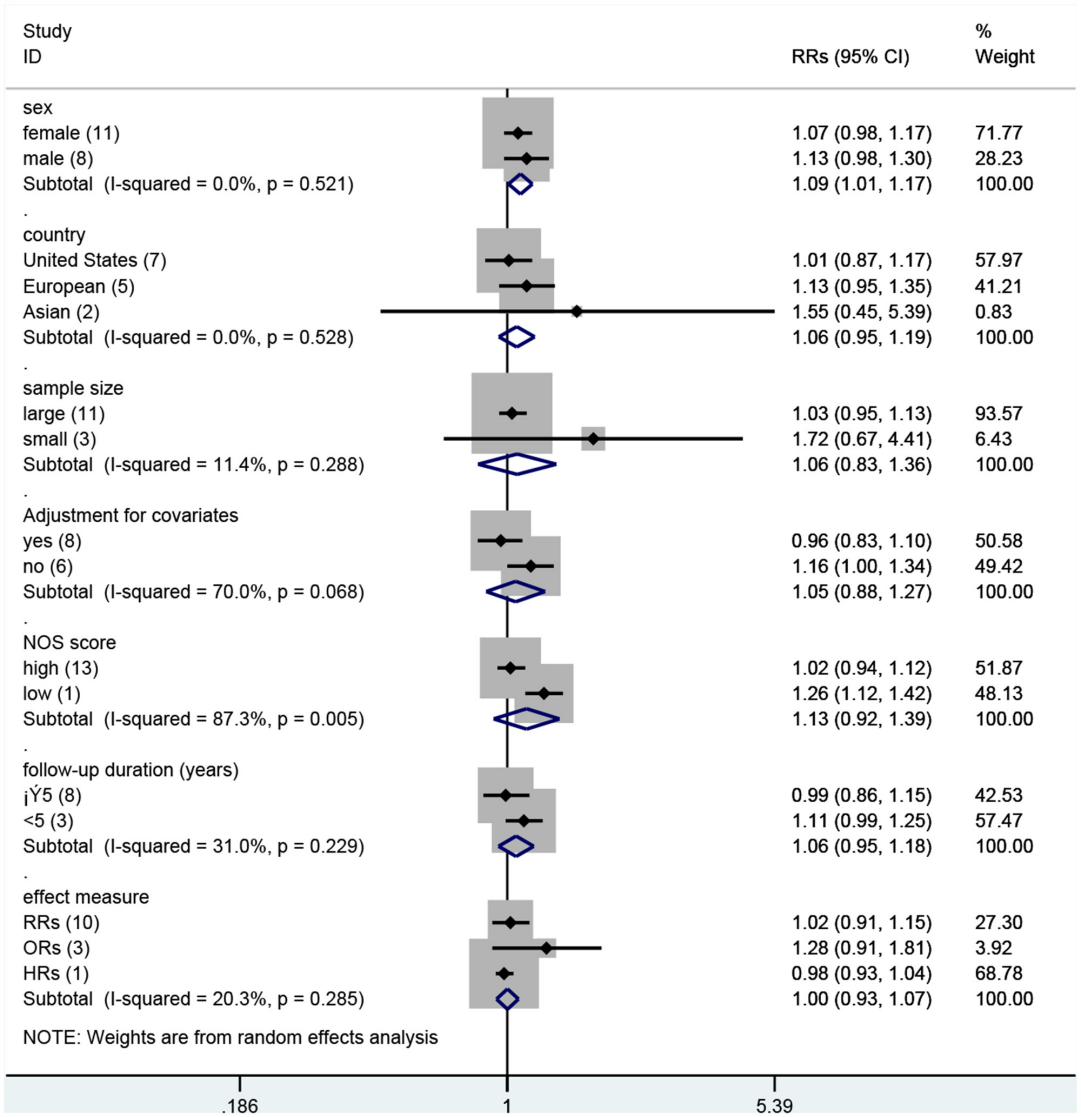
Sensitivity analyses based on various exclusion criteria.

To explore potential heterogeneity, we performed sensitivity analyses to examine the effects of various exclusion criteria on combined risk estimates. Sensitivity analyses based on adjustment for covariates, effect measure, country, sex, sample size (Large, cases ≥ 100; Small, cases <100), quality of Newcastle—Ottawa Scale scores (High, NOS score ≥5, Low, NOS score, <5), and follow-up period yielded similar results with substantial evidence of heterogeneity (Table 4, Fig 4). Further exclusion of any single study did not significantly alter the combined RRs, which ranged from 1.02 (95% CI = 0.94 to 1.12) to 1.08 (95% CI = 0.99 to 1.17).

**Table 4:** Sensitive analyses based on various exclusion criteria.

	Studies,N	RRs,95%CIs	P value	P value for heterogeneity	I^2^(%)
total	14	1.05(0.96–1.15)	0.291	0.000	82.8
Country					
United States	7	1.01(0.87–1.17)	0.898	0.000	84.1
Europe	5	1.13(0.95–1.35)	0.168	0.001	79.7
Asia	2	1.55(0.45–5.39)	0.491	0.012	84.3
Gender					
Female	11	1.07(0.98–1.17)	0.143	0.000	83.5
Male	8	1.13(0.98–1.30)	0.167	0.000	86.5
Effect measure					
RRs	10	1.02(0.91–1.15)	0.712	0.000	83.9
ORs	3	1.28(0.91–1.81)	0.162	0.087	59.0
HRs	1	0.98(0.93–1.04)	0.479	NA	NA
Sample size					
Large(cases ≥ 100)	11	1.03(0.95–1.13)	0.451	0.000	84.5
Small(cases <100)	3	1.72(0.67–4.41)	0.263	0.078	60.9
Adjustment for covariates					
Yes	8	0.96(0.83–1.10)	0.529	0.000	75.0
No	6	1.16(1.00–1.34)	0.050	0.000	89.4
NOS score					
High(≥5)	13	1.02(0.94–1.12)	0.600	0.000	80.8
Low(<5)	1	1.26(1.12–1.42)	0.000	NA	NA
Follow-up duration(years)					
≥5	8	0.99(0.86–1.15)	0.978	0.000	83.0
<5	3	1.11(0.99–1.25)	0.075	0.000	87.1
NA	2	1.64(0.52–5.16)	0.394	0.030	78.8

HRs, hazard ratios, ORs, odds ratios, RRs, relative risks, CIs, confidence intervals; NA, not available.

### Meta-regression

Considering the relatively high heterogeneity exhibited in the trials, a meta-regression was conducted to explore the predefined possible sources of heterogeneity. None of the regression coefficients were statistically significant (Table 5), suggesting that country, gender, quality of Newcastle—Ottawa Scale scores, adjustment for covariates and sample size were insignificant sources of heterogeneity.

**Table 5:** Effects of study variables by meta-regression.

Covariant	Coefficient	P value	95% CIs
Country(Ref = Europe)			
Asia	−0.004	0.991	−0.740 to 0.732
United States	−0.161	0.475	−0.640 to 0.318
Adujusted for covariates	−0.243	0.180	−0.615 to 0.129
Sample size	−0.633	0.115	−1.445 to 0.179
Newcastle—Ottawa Scale scores	−0.051	0.451	−0.193 to 0.091
year of publication	-0.014	0.276	−0.039 to 0.012

### Publication bias

**Fig 5:**
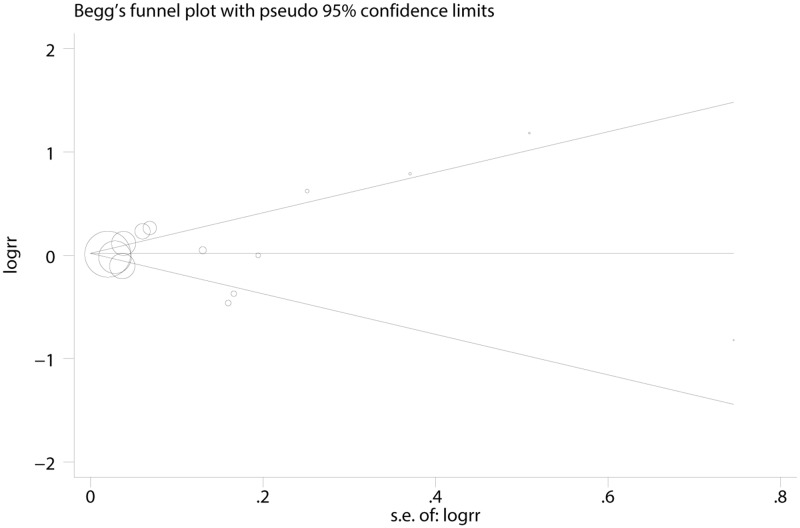
Funnel plots of exposure to fluoride in the drinking water and hip fracture risk for assessment of publication bias.

Both Begg’s (rank correlation test) and Egger’s funnel plot asymmetry test (regression method) in the meta-analysis indicated no significant publication bias (Begg’s test, P = 0.74; Egger’s test, P = 0.47; Fig 5).

## Discussion

To our knowledge, this meta-analysis is the first to explore the effect of fluoride exposure on hip fracture patients. The pooled results from the meta-analysis of 14 observational studies using random-effects model provided evidence that chronic fluoride exposure from drinking water does not significantly increase the incidence of hip fracture. The combined estimates were robust across sensitivity and meta-regression. Few evidence of publication bias was also observed.

Several plausible mechanisms may explain the lack of association between fluoride exposure from drinking water and hip fracture risk in the present analysis. Fluoride is a bone seeker and has strong effects on bone cell function, bone structure, and bone strength [21]. Fluoride can affect bone in at least two ways. Fluoride ions can replace hydroxyl ions in the hydroxyapatite lattice, thereby increasing fluoride concentrations in plasma and osteoblastic differentiation and activity. Such changes play a crucial effect on fracture risk. This effect has only been observed when intake has been substantially higher than expected from water fluoridation. However, the implications of lower exposure for fracture risk are uncertain.

The effects of fluoride on bone have also been investigated in a randomized controlled trial of high-dose sodium fluoride (75 mg daily) as a treatment for osteoporosis. During a four-year period, the intervention led to a substantial increase in bone density in the spine [22]. Although this study indicated a hazard from high doses of fluoride in people with established osteoporosis, the risk cannot be necessarily extrapolated to much lower doses received by the general population from fluoride in water supplies.

Danielson et al. [7], Kurttio et al. [5], and Sowers et al. [4] reported that exposure to fluoride could increase hip fracture risk among older women, whereas Cauley et al. [9] and Phipps et al. [8] indicated that fluoride exposure slightly decreases hip fracture risk among older women. Jacobsen et al. [14], Karagas et al. [11], Näsman P et al. [10], and Park et al. [13] have found no association. In the present meta-analyses, no statistically significant association between fluoride exposure from drinking water and hip fracture risk was observed among older women. In older ages, several reasons account for fluoride effects. One explanation could be the effect of calcium. Calcium supplementation is usually recommended for elderly patients. However, excess dietary calcium intake may prohibit fluoride absorption, which reduces probability of fluoride effect [23]. Calcium absorption is also different in elderly patients, which decreases with age as a result of various possible mechanisms, including decreased vitamin D intake, synthesis, and metabolism [24]. Elderly persons also tend to reduce their food intake, which further decreases their opportunity for adequate nutrition. The kinetics of fluoride may be different in older ages [25]. The rate of bone formation decreases as the skeleton ages, and less fluoride is taken up by older bone [21], thereby reducing potential effects of fluoride.

The present meta-analysis has some limitations. First, our meta-analysis was based on observational studies, and half of the included studies controlled some confounding factors, such as age and gender. However, other confounding factors (estrogen level, total calcium intake, diet, and level of fluoride exposure) are difficult to control in epidemiological studies. Second, although few evidence of publication bias was observed, the statistical power for these tests was limited because of the relatively small number of included studies. Third, significant heterogeneity was detected in our meta-analysis. Heterogeneity between studies should not be ignored even if it is highly common in the meta-analysis. We also performed sensitivity analyses and meta-regression to determine the sources of heterogeneity, but heterogeneity was still observed. Fourth, because the majority of studies using different methods used to assess and categorize fluoride exposure among studies (Table 1), our findings are likely to be influenced by the misclassification of exposure. In cohort studies, this misclassification would likely be non-differential if the exposure variable was dichotomous, and thereby result in an underestimate of the true association, whereas the influence of a misclassification on the results in case-control studies is less predictable. Moreover, the potential for misclassification of exposure to fluoride may contribute to the heterogeneity for all studies in the summary analysis. Therefore, this result should be considered with caution because of exposure misclassification. Overall, these aforementioned limitations may affect our final conclusions.

In conclusion, limited evidence suggests that chronic fluoride exposure from drinking water does not significantly increase the risk of hip fracture. Although these findings are encouraging, the results of this meta-analysis should be explained with caution because of potential confounding factors, heterogeneity, and exposure misclassification. Further large-scale and well-designed trials on this topic are needed.

## Supporting Information

S1 PRISMA Checklist (DOC)
